# Cross-Species Exome Sequencing Reveals Recurrent Genomic Alterations in California Sea Lion (*Zalophus californianus*) Urogenital Carcinoma and Highlights a Recurrent PD-L1 Variant

**DOI:** 10.3390/genes17020222

**Published:** 2026-02-10

**Authors:** Isabella G. Livingston, Catherine F. Wise, Allison N. Dickey, Rachael Thomas, Alissa C. Deming, Barbie Halaska, Frances M. D. Gulland, Kathleen M. Colegrove, Pádraig Duignan, Matthew Breen

**Affiliations:** 1 Department of Molecular Biomedical Sciences, College of Veterinary Medicine, North Carolina State University, Raleigh, NC 27607, USA; igliving@ncsu.edu (I.G.L.);; 2 Bioinformatics Research Center, North Carolina State University, Raleigh, NC 27607, USA; 3 Center for Human Health and the Environment, North Carolina State University, Raleigh, NC 27607, USA; 4 Comparative Medicine Institute, North Carolina State University, Raleigh, NC 27607, USA; 5 Department of Conservation Medicine and Science, Pacific Marine Mammal Center, Laguna Beach, CA 92651, USA; 6 The Marine Mammal Center, Sausalito, CA 94965, USAfrancesgulland@gmail.com (F.M.D.G.); duignanp@tmmc.org (P.D.); 7 School of Veterinary Medicine, University of California, Davis, CA 95616, USA; 8 Zoological Pathology Program, Veterinary Diagnostic Laboratory, College of Veterinary Medicine, University of Illinois, Brookfield, IL 61802, USA

**Keywords:** marine mammals, comparative oncology, comparative genomics, non-model organisms, wildlife cancer, sentinel species, California sea lion, urogenital carcinoma, PD-1/PD-L1 pathway

## Abstract

Background/Objectives: Human-driven environmental change can promote cancer development in wild species, yet the pathophysiology of wildlife cancers remain largely unexplored. Urogenital carcinoma (UGC) in the California sea lion (CSL) (*Zalophus californianus*) is one of the most common cancer types documented in any wild mammal. The pathogenesis of UGC in CSLs is known to be multifactorial, with links to environmental contaminant exposure and infection by Otarine Herpesvirus-1 (OtHV-1); however, the genomic features of these cancers have not been thoroughly explored. Understanding UGC pathogenesis in the CSL has important implications for the health of humans and other species that share environment and diet. Methods: We leveraged the evolutionary conservation between the domestic dog and CSL genomes to perform cross-species whole-exome sequencing (WES) of CSL UGC tumors and matched normal tissue pairs. We also used PCR and Sanger sequencing to investigate the prevalence of DNA from OtHV-1. Results: Bioinformatic analyses identified shared somatic variants and DNA copy number aberrations in UGC tumor samples, including recurrent exonic single-nucleotide variants in *CD274/PD-L1*, and recurrent copy number gains in *CD274/PD-L1*, *TNFRSF14*, *CD200*, *CDK4*, and *PLCG2*. In an extended cohort of 70 CSLs (tumor, matched normals, and controls), a recurrent C > T single-nucleotide variant in exon 4 of *CD274/PD-L1* was identified in 54 of 68 (79.4%) CSLs with diagnosed UGC. OtHV-1 DNA was detected in 67 of 70 individuals (95.8%). Conclusions: These results demonstrate that cross-species exome capture provides a means to identify genomic alterations that may play a role in the molecular pathogenesis of UGC in the CSL and adds to the body of evidence for an association between OtHV-1 and UGC in this species.

## 1. Introduction

Humans are increasingly recognized as an oncogenic species or agent, meaning that human-induced environmental changes can drive cancer development in animal populations [1,2,3]. Anthropogenic activities have led to an increased incidence of cancer across taxa through the release of environmental pollutants and environmental modulation [1,4,5,6]. While cancers in wildlife have become an increasing concern, they remain understudied [1,7,8]. The link between exposures to environmental contaminants and the development of neoplasia in wild species mirrors patterns noted in humans, with cancer largely associated with aspects of human ecology, such as infections and exposures to mutagens [7,9,10]. Notably, elevated cancer risk in wildlife is found most often in populations residing in polluted habitats or those exposed to oncogenic pathogens, suggesting a parallel between human and wildlife cancer epidemiology [7]. These similarities underscore the importance of studying spontaneous cancer in wildlife, not only to safeguard the survival of affected species but also to enhance our understanding of the etiology and pathogenesis of cancer in humans and to reveal evolutionarily conserved mechanisms that contribute to disease development [11,12].

Urogenital cancers, in general, comprise a variety of malignancies of the urinary and genital systems, such as those of the bladder and renal systems, prostate, testicles, ovaries, uterus, and cervix [13,14]. In humans, these cancers represent a significant portion of the global cancer burden, and in the United States they are reported to account for one in five new cancer cases and one in seven cancer-related deaths each year [6,15]. The California sea lion (CSL) (*Zalophus californianus*) is a marine mammal that has a breeding range in southern California and is seasonally distributed along the west coast of North America. The CSL is remarkable in that it exhibits a high incidence of urogenital carcinoma (UGC), representing one of the highest prevalences of a single type of cancer reported in any mammalian species [16,17,18]. In sea lions, UGC mainly affects adults and originates primarily in the cervix and/or vagina and uterus of females and the penis and/or prepuce of males, followed by metastases to secondary sites, including lymph nodes, kidney, urinary bladder, liver, lungs, spleen, peritoneum, omentum, and muscle, ultimately causing death of the affected individual [16,19,20,21]. Over the past four decades, up to 26% of adult CSLs examined post-mortem at The Marine Mammal Center (TMMC) in Northern California have been diagnosed with UGC [2,16,17,22,23]. Studies have revealed a range of factors associated with the development of this disease, including viral infection, environmental contaminants, and genetic predispositions [2,16,17,21].

A novel gammaherpesvirus, Otarine herpesvirus-1 (OtHV-1), has been identified in the CSL and is hypothesized to have a significant association with UGC development [17,18,24,25]. OtHV-1 is genetically similar to Epstein–Barr virus (EBV) and Kaposi sarcoma-associated herpesvirus (KSHV), both oncogenic herpesviruses in humans [2,17,20,21,26]. Worldwide, approximately 120,000 and 40,000 annual cases of new human cancer diagnoses are attributable to EBV and KSHV, respectively [27]. Though the association between viral infection and cancer is well-supported in the CSL and other species, including humans, additional cofactors or exposures are required for neoplasia development [20,25,27]. Notably, CSLs are exposed to elevated levels of environmental contaminants, including halogenated organic compounds (HOCs), polychlorinated biphenyls (PCBs), and other persistent organic pollutants, due to their coastal habitat and top trophic position [2,17,28,29]. Studies have reported that mean blubber concentrations (based on wet weight) of PCBs and dichlorodiphenyltrichloroethane (DDT) in CSLs with cancer are 85% and 30% higher, respectively, than in CSLs without cancer [16,25]. Certain classes of environmental contaminants are known to induce somatic mutations and other forms of DNA damage, promote cell proliferation, and/or impair immune responses, mechanisms that may contribute to increases susceptibility to infection by oncogenic viruses and promote tumor development [1,10,25,30,31]. This suggests that high levels of contaminant exposure, in conjunction with OtHV-1 infection, may contribute to the increased incidence of cancer in the CSL [1,2,25].

The scarcity of information on the pathology of wildlife cancer, coupled with the high incidence of UGC in CSLs, positions this species as a valuable model for neoplasia research [11]. CSLs serve as valuable indicator species for ecosystem health due to their long lifespan, high trophic positions, and blubber stores that sequester lipophilic agents [19,21,32,33]. Humans and CSLs develop cancers spontaneously and share environmental exposures and food sources [16,17,33]. The solidified links between animal and human health underscore the importance of studying species that develop cancer due to anthropogenic influence. Such species can act as sentinels for understanding the evolutionarily conserved mechanisms underlying cancer development as well as the risks to both wildlife and humans [2,5,34,35].

Contaminants linked to UGC in the CSL have been associated with cancers in humans [7,36], and CSL UGC displays a similar pathology to human cancers, such as cervical cancers [2,5,17,37]. In both species, a synergistic relationship between pollutants and oncogenic viruses is thought to drive cancer development, with approximately 15% of human cancer diagnoses having an association with an infectious agent [27,38,39]. Endocrine-disrupting HOCs are suspected to be major contributing factors in the development of human hormone-dependent malignancies, including breast, testicular, and prostate cancers [10,36], and oncogenic viruses show strong associations with increased risks of human cervical cancer, lymphomas, penile cancers, and liver cancer [38,40]. Domestic dogs have been evaluated in this capacity for decades, revealing conserved patterns of recurrent genomic changes between canine and human cancers [5,12,41,42,43,44,45,46,47,48,49,50,51,52,53,54]. Expanding comparative genomic studies to include additional species, like the CSL, will strengthen our ability to identify key drivers of pathogenesis that are species agnostic.

Though it is known that the causes of UGC development in the CSL are multifactorial, the genomic characteristics of these neoplasms remain understudied [24]. An improved understanding of cancer pathogenesis in the CSL will create opportunities to explore potential implications for the health of humans and related species that share the environment and resources. To address this, we leveraged the genome sequence similarities between the domestic dog and CSL, both members of the suborder Caniformia [55], to generate whole-exome sequence (WES) data from CSL tumors and grossly unaffected patient matched tissue samples.

Exome capture kits generally comprise a pool of synthetic DNA sequences (capture baits) designed to be complementary to the exon sequences of the target species. While they are species-specific in design, sufficient sequence homology between species allows cross species use. For example, exome capture kits designed for human genomes have been used to sequence Neanderthal exomes [56] and kits designed for domestic cattle have been deployed to capture related bovid species [57]. While canine exome capture baits have previously been used for studies of other extant members of the Canidae (spanning ~10–12 M yrs of speciation), here we used canine baits to capture exon sequences from the CSL, estimated to have diverged from a common ancestor during the Eocene epoch, ~40–50 million years ago [58,59]. Using the captured exon sequences, we investigated genomic alterations associated with UGC in the CSL. This cross-species approach identified recurrent somatic variants in the genomes of CSL UGC tumors from various primary and metastatic sites, providing new insight into the conserved pathogenesis of this disease.

## 2. Materials and Methods

### 2.1. Sample Acquisition and Histopathology

CSLs that were stranded, sick, or injured along the California coast, were rescued and taken for veterinary assessment and care to the Marine Mammal Center California (TMMC, Sausalito, CA, USA; MMPA permit 18786) or the Pacific Marine Mammal Center (PMMC, Laguna Beach, CA, USA; all activities were conducted under Stranding Agreement between PMMC and NOAA). CSLs included in this study died or were humanely euthanized and were histologically diagnosed with either UGC (cases) or other non-cancer diseases (controls).

Necropsy: At necropsy for both UGC cases and controls, the entire reproductive tract was resected and uroepithelium examined and photographed before subsampling for genomics (see below), followed by fixation of the remaining tract in 10% neutral buffered formalin. In addition, samples of all major organs were formalin-fixed, including multiple lymph nodes, spleen, liver, lung, kidney, heart, digestive tract, skeletal muscle, endocrine organs, central nervous system, eyes, integument and, for some cases, bone. Fixed tissues were trimmed in by a veterinary pathologist or skilled histotechnologist and processed for hematoxylin and eosin (H&E) staining at an accredited laboratory (Anatomic Pathology, University of California, Davis or University of Illinois). For female CSLs, tissues for histologic assessment included cervix, vagina, uterine horns, and ovaries, while for males, the glans penis and multiple sections of prepuce were examined. For both sexes, the urinary bladder at the apex and trigone, urethra, and the most common sites for metastasis such as inguinal and sublumbar lymph nodes, lungs, spleen, kidneys, adrenal glands and liver, were also examined. For each case, representative grossly visible lesions were trimmed in for histologic evaluation. CSLs were classified as controls if there was no histologic evidence of UGC in the cervix/vagina/uterus or penis/prepuce and no evidence of metastases. Primary disease processes identified in these individuals are listed in Table 1. All other CSLs had carcinoma identified histologically arising from the cervix/vagina and in some cases uterus, or penis/prepuce, as described in previous studies [60]. UGC lesions were categorized as either (1) intraepithelial or carcinoma in situ when limited to the uroepithelium of the cervix/vagina or penis/prepuce, (2) invasive when there was distinct invasion beneath the epithelial basement membrane into the underlying stroma, or (3) metastatic when there was evidence of intravascular invasion and/or metastases in regional lymph nodes or in other organs distant from the primary tumor. Case-specific details for the eight individuals used in WES are shown in Supplementary Table S1.

Sampling for genomics: During the necropsy, a 3 × 2 × 1 cm piece of each tumor observed, and a matched piece of grossly normal tissue (distant muscle), were placed in separate 50 mL vials containing 40 mL RPMI-1640 medium (Gibco, Waltham, MA, USA) supplemented with 2 mM Glutamax (Gibco, Waltham, MA, USA), 100 μg/mL Primocin (InvivoGen, San Diego, CA, USA), and 10 mM HEPES (Gibco, Waltham, MA, USA). For two individuals (CSL 13281 and 14669), multiple unaffected muscle samples from distinct anatomical sites were available and were included as biologically independent normal tissues; these samples were processed separately and accounted for at the individual level in downstream analyses, such that individuals were not weighted by the number of samples contributed. For controls (no evidence of UGC), samples were collected from the cervix, lung, and muscle. For some individuals, whole blood was collected in place of or in addition to unaffected tissue. These specimens were placed on ice and shipped overnight to the North Carolina State University College of Veterinary Medicine. In total, 171 samples were collected from 70 CSLs (91 tumor samples, 76 matched normal tissue or blood samples, 4 tissue or bloodsamples from non-UGC controls) (Table 1(a,b)). On arrival, each tissue sample was photographed and a ~25 mg section removed for DNA isolation. For seven individuals, a similarly sized piece of tissue was exposed to collagenase type II at 37 °C overnight, and the resulting cell suspension was used to establish a primary cell culture. The remainder of each tissue specimen was stored at −80 °C.

### 2.2. DNA Extractions

DNA was extracted from all tumor and non-tumor tissue samples using the Qiagen DNeasy Blood and Tissue Kit (Qiagen, Valencia, CA, USA) per the manufacturer’s recommendations. DNA samples were assessed for integrity, quantity, and purity via a combination of agarose gel electrophoresis and spectrophotometry (Nanodrop^®^ ONE, Thermo Fisher Scientific, Wilmington, DE, USA; 260:230 > 2.0 and 260:280 > 1.8). All DNA extracts were stored at −20 °C pending analysis.

### 2.3. OtHV-1 Detection

DNA derived from all 171 CSL specimens (tumors and matched-normals, plus tissue from non-cancer controls) was screened using a published PCR protocol targeting unique areas of the OtHV-1 DNA-dependent DNA polymerase sequence (300 bp) [61]. This and all subsequent PCRs were performed on a Bio-Rad T100 thermocycler (Bio-Rad, Hercules, CA, USA) and evaluated via agarose gel electrophoresis for the presence of the expected amplicon. Amplicons were submitted to the North Carolina State University Genomic Sciences Laboratory (NCSU GSL) for bidirectional Sanger sequencing. The resulting data files were used for taxonomic analysis with BLAST 2.17.0 (NCBI), using percent identity and E-values to determine positive matches.

### 2.4. Whole-Exome Sequencing

In the absence of species-specific reagents, we leveraged the DNA sequence similarity between the genomes of the CSL and domestic dog to identify somatic SNVs and CNAs in the CSL tumors, using canine WES baits (Roche NimbleGen, Madison, WI, USA, 120705_CF3_Uppsala_Broad_EZ_HX1). From our collection of CSL UGC tumor and matched normal (muscles) samples, eight were selected for WES based on their highly aberrant DNA copy number profiles, as determined previously using oligonucleotide array comparative genomic hybridization analysis [62]. The eight paired samples (six female and two male) comprised one primary cervical tumor and seven metastatic tumors from liver (*n =* 3), bladder (*n* = 2), kidney (*n* = 1), lymph node (*n* = 1). Genomic DNA specimens were sheared acoustically to mean fragment sizes of 300 bp using a Covaris-Focused ultrasonicator (Covaris, Woburn, MA, USA). Library preparation was performed using the KAPA Hyper Prep Kit following the SeqCap EZHypercap workflow, following the manufacturer’s instructions (Kapa Biosystems, Wilmington, MA, USA). Per suggestion from Roche NimbleGen, SeqCap EZ Developer Reagent (Roche NimbleGen, Madison, WI, USA) was used in place of CoT-1 competitor DNA. The indexed libraries were submitted for 150 bp paired-end sequencing on an Illumina NovaSeq 6000 platform (NCSU GSL) with target coverage of 100× for the tumors and 30× for the normals.

The reads were trimmed using fastp (v 0.21.0) (default settings) [63] and then mapped to the CSL genome assembly, mZalCal1.pri.v2 (GCF_009762305.2), using bwa mem -M (v 0.7.17) [64]. The alignment files were processed through the Picard (v 2.25.6) CleanSam tool with the VALIDATION_STRINGENCY = LENIENT argument. Read group information was added using the Picard AddOrReplaceReadGroups tool, samtools (v 1.12) [65] was used to coordinate-sort the alignment files, and the Picard MarkDuplicates tool was used to identify duplicate reads.

Mutect2 (GATK v 4.2.0.0) [66] was used to identify somatic SNVs for the tumor/normal pairs, and the command was run with the—f1r2-tar-gz argument. The GATK LearnReadOrientationModel tool was used to create an artifact prior table from the F1R2 metrics. Filters were applied to the Mutect2-generated VCF files using the GATK FilterMutectCalls tool, which included an argument for the orientation bias artifact priors. The variants that passed these filters were saved to a separate VCF file using bcftools (v 1.13) [65].

Using liftOver 1.3.3 [67], the genome coordinates of all canine exome bait sequences were converted to their corresponding location coordinates in the CSL genome. Following conversion, samtools bedcov was used to calculate the coverage, number of bases sequenced above a specified depth threshold (30× for the normal, 100× for tumors), and the read counts across all regions in the exome bed file. The resulting files were read into RStudio (v.23.3.0) and used to calculate the mean and median coverage, percentage of bases above the specified depth threshold, and percentage of bases covered at least once. These metrics were calculated both on a per-sample and per-chromosome basis. The data were plotted using the ggplot2 RStudio package (v.3.4.0) [68].

Gene-level orthology assignments were determined by comparing annotated gene identities at bait target sites using the NCBI RefSeq GFF annotations for dog (GCF_000002285.3; CanFam3.1) and CSL (GCF_009762305.2), downloaded August 2024. Regions where dog baits targeted exons annotated with the same gene symbol in both species were classified as ‘high orthology’, while regions where gene annotations differed were classified as ‘reduced orthology’, and regions with no gene annotation overlap were classified as ‘no interpretable orthology’.

### 2.5. Variant Validation

SNVs were filtered to include only those located within an exon of a RefSeq-annotated CSL gene (GCF_009762305.2 annotation release 101). A candidate list of variants was compiled, comprising alterations shared by two or more of the eight CSL tumor samples, with variant allele frequencies ≥10%. Amplicon sequences from the CSL reference genome (GCF_009762305.2_mZalCal1.pri.v2) containing the variant regions were downloaded from the UCSC Genome Browser (genome.ucsc.edu) and uploaded to IDT PrimerQuest (Integrated DNA Technologies, Coralville, IA, USA). Primer sets were tested using CSL genomic DNA from one male (#10376) and one female (#13276) pair of tumor and normal muscle as templates for endpoint PCR. PCR amplification was performed with the following reagents: 10 μL 2× RedTaq MasterMix (Sigma-Aldrich, St. Louis, MO, USA), 1 μL each of the 10 μM forward and reverse primers, 7 μL of water, and 1 μL (10–25 ng) of template DNA. One microliter of nuclease-free water was used in place of template DNA for a no-template control that was run in parallel with the CSL samples. Following amplification, PCR amplicons were visualized on a 2% agarose gel and submitted to the NCSU GSL for bidirectional Sanger sequencing using the PCR primers. The primer set for each variant that produced the cleanest sequence trace was used to test the entire WES sample cohort under the same PCR reaction conditions. For validated variants, an additional 62 CSL individuals (*n* = 83 tumors, *n* = 72 matched/control normal tissues or blood) were evaluated by PCR and Sanger sequencing.

### 2.6. DNA Copy Number Analysis

The deduplicated BAM files for each of the eight CSLs in the WES cohort were used for DNA CNA analysis using GATK4 [69,70]. Intervals of 5 kb along each CSL autosome and the X chromosome were compiled from the CSL reference genome assembly (GCF_009762305.2_mZalCal1.pri.v2) with the PreprocessIntervals command. These intervals were then used to collect raw read count data from each deduplicated BAM file with the CollectReadCounts command. The read counts from each tumor were denoised against their matched normal sample (matched comparison) using the DenoiseReadCounts tool to generate a tsv file with log2 DNA copy ratio values.

The resulting denoised tsv file for each tumor sample was used to construct DNA copy number plots. First, the log2 copy ratio value was used to calculate the copy number ratio for each tumor sample. These values were then plotted using ggplot2 [68] to visualize the copy number ratios along each autosome and the X chromosome. Regions of copy number gains (copy number ratio > 1) and copy number losses (copy number ratio < 1) were extracted for each CSL tumor, then assessed to identify shared CNA regions across all samples. For all shared regions, the UCSC Genome Browser was used to determine if they fell within exons of an annotated CSL gene.

## 3. Results

### 3.1. OtHV-1 Detection

**Figure 1:**
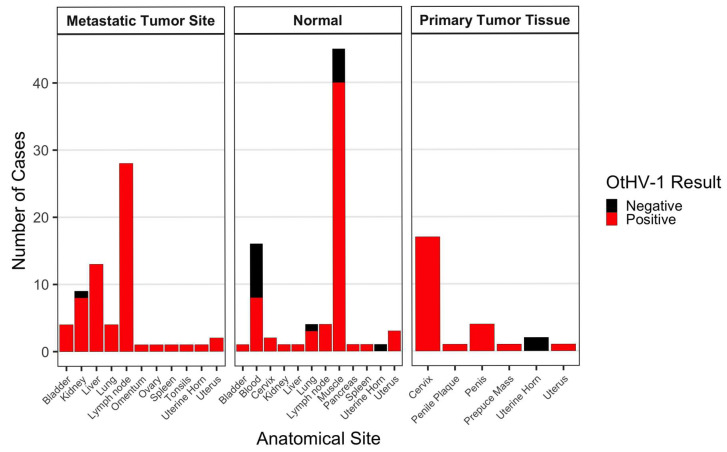
Detection of OtHV-1 DNA in CSL samples. Samples were classified as normal tissue, primary tumor tissue, or metastatic tumor tissue sites. All samples (*n* = 171; 91 tumors, 80 normals) representing 70 CSLs were screened by OtHV-1 PCR. Sixty-seven (95.7%) of the 70 individuals tested, including one control, had at least one sample positive for OtHV-1 DNA.

PCR screening and subsequent bidirectional Sanger sequencing revealed a high prevalence in our CSL cohort (Figure 1). All positive amplicons showed >99% sequence identity to the OtHV-1 reference sequence (GenBank accession: AF236050.1) with E-values < 1 × 10^−50^, and with no matches to other herpesvirus species, and were consistent with species-specific identification.

Of the 70 individual CSLs included in this study, 67 (95.7%) had at least one sample (tumor or matched normal) positive for OtHV-1 DNA. Sixty-four of the 70 individuals were confirmed to have metastatic UGC, and of these, 62 individuals (96.9%) tested positive for OtHV-1 DNA in one or more tumor samples. Two individuals with confirmed UGC had no detectable OtHV-1 DNA in any tested sample.

Sixty-one individuals (*n* = 60 metastatic UGC, *n* = 1 carcinoma in situ (CIS)) had both tumor and matched normal samples tested. Fifty of these individuals were positive in all samples tested (82.0%). One individual was negative in all samples (1.6%). Nine of these CSLs showed discordant patterns, where tumor samples were positive but matched normal samples were negative (14.8%), and one individual had two matched normal samples with discordant results: tumor and muscle tissue tested positive while blood tested negative (1.6%). No cases had a positive normal sample without a positive tumor sample. Among the nine discordant cases (tumor-positive/normal-negative), five (55.6%) had blood-derived normal samples and four (44.4%) had tissue-derived normal samples.

Seven of the 68 CSLs with UGC had only tumor samples tested (no matched normal available). Of these, six had primary tumors positive for OtHV-1, including three CIS. One of these individuals had both a primary and metastatic tumor tested, and only the primary was positive while the metastasis was negative, representing the only instance of intra-individual tumor heterogeneity in viral detection in this cohort. OtHV-1 DNA was detected in two of four samples obtained from non-UGC CSLs; however, positive detections were restricted to blood or urogenital mucosal tissues (cervix). The single control individual without detectable OtHV-1 was represented only by lung and muscle tissues, which are not established sites of herpesvirus latency.

OtHV-1 DNA was detected significantly more frequently in tumor samples than in non-tumor tissues (Fisher’s exact test, *p* = 0.001, OR = 6.70). This enrichment remained significant when tumor tissues were compared specifically to matched normal tissues from affected animals (*p* = 0.003, OR = 6.0). Mixed-effects logistic regression accounting for multiple tissues per individual confirmed a strong association between tumor status and viral detection (*p* < 0.001).

### 3.2. Whole-Exome Sequencing

**Figure 2:**
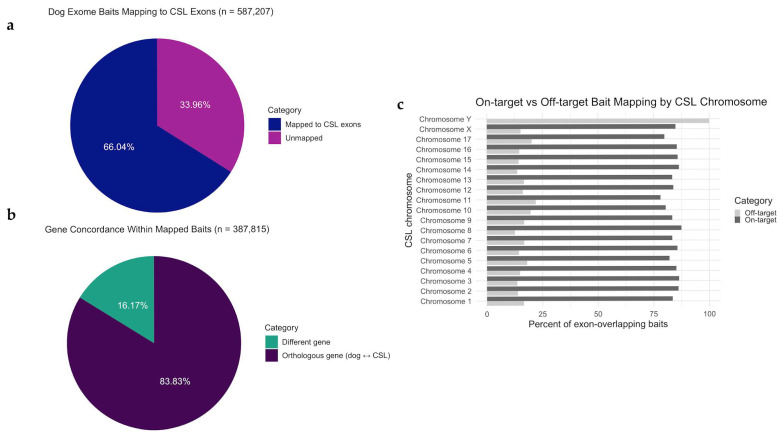
Mapping efficiency of canine exome capture baits to the CSL reference genome. (**a**) Total percent of dog baits mapping to CSL exons. (**b**) Gene concordance of baits that mapped to CSL exons. (**c**) Percent on and off-target baits mapping to each CSL chromosome. On-target is defined by the percent of baits per chromosome that mapped to an exon of the orthologous gene in both the domestic dog and the CSL. Off-target denotes the number of baits that map to a CSL exon but do not map to the orthologous gene in the domestic dog.

To evaluate the performance of the canine exome capture system in the CSL, we first assessed how the baits (*n* = 587,207) comprising the NimbleGen SeqCap EZ canine set computationally aligned to the annotated CSL genome assembly (GCF_009762305.2). In total, 518,656 dog capture baits (88.3%) successfully lifted to the CSL genome, and 66.04% (*n* = 387,815) aligned with predicted CSL exons (Figure 2a). The remaining 130,841 baits that did not align with annotated CSL exons mapped to intergenic regions or unannotated sequences.

Notably, 83.8% (*n* = 325,111) of the 387,815 baits mapped to the same gene in both species (Figure 2b). These data demonstrate that 55.4% of the total canine exon baits had strong preservation of gene-level orthology in the CSL. The 14.6% of canine baits that mapped to exons of non-orthologous genes between the two species likely reflect a combination of chromosomal rearrangements, gene duplications or deletions, or assembly artifacts that have occurred since the divergence of Canidae and Otariidae, and highlight regions where synteny may be disrupted or where paralogous gene families have evolved differently between the two lineages.

**Figure 3:**
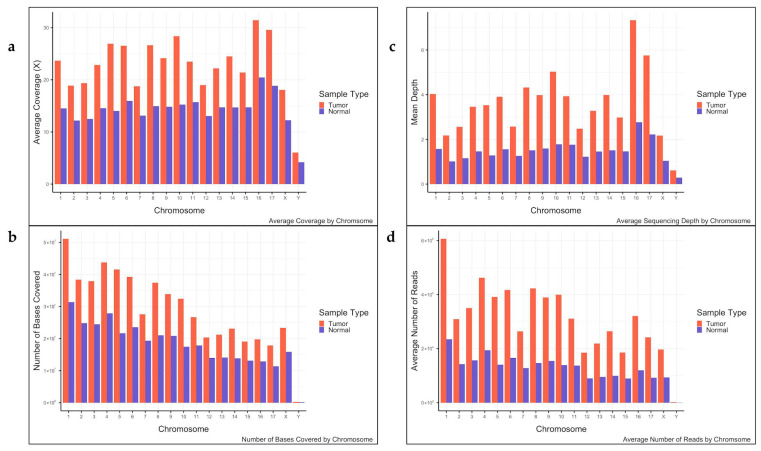
Sequencing summary statistics for the eight CSL tumor-normal pairs analyzed by WES. Per-chromosome statistics include (**a**) average per-base coverage, (**b**) mean sequencing depth, (**c**) number of bases covered, and (**d**) average number of reads.

Cross-species exome sequencing results aligned with the expected depth differences between tumor and normal tissues. Tumor DNA achieved an average per-base coverage of 106.2 ± 0.3× (standard error), compared to 41.2 ± 0.1× for paired normal DNA, producing a tumor-to-normal depth ratio of 2.58:1×. Exome capture efficiency was high, with an average of 77.7% of tumor reads and 75.8% of normal reads mapping to annotated CSL exons (range: 73.1–83.9% for tumor samples, 63.2–82.5% for normals). Target enrichment was efficient, with 98.2% of captured bases from tumor samples and 96.9% of captures bases from normal samples covered at least once (T:N 1.01:1). Depth distributions showed that on average, 36.5 ± 0.1% of tumor DNA bases and 47.5 ± 0.1% of normal DNA bases exceeded their respective depth thresholds (≥100× tumor; ≥30× normal) (Figure 3). Per-individual and per-chromosome metrics are summarized in Supplementary Tables S2 and S3, respectively.

### 3.3. Variant Validation

**Figure 4:**
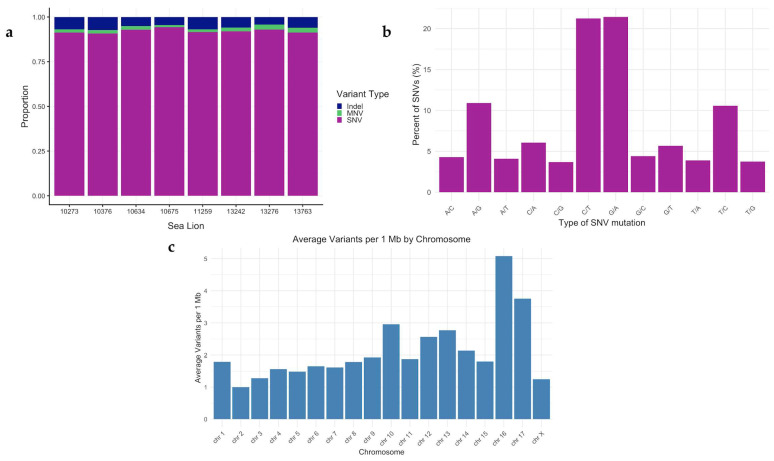
Somatic variant identification across WES tumors. (**a**) Proportion of variant types for all eight tumors subjected to WES, (**b**) Percent frequency of each SNV type identified by Mutect2, and (**c**) Average number of somatic variants per 1 Mb by CSL chromosome.

Across all CSL tumor samples, 4652 SNVs, 298 indels, and 196 MNVs were identified (Figure 4a). Looking at the type of SNVs present revealed a predominance of C > T and G > A transitions, which together accounted for approximately 42% of all single-nucleotide changes (Figure 4b). When normalized by chromosome size, the variant density ranged from approximately 1.0 to 5.1 variants per Mb across the 17 CSL autosomal chromosomes. Chromosomes 16 and 17 exhibited notably higher variant densities (5.1 and 3.7 variants per Mb, respectively), which may reflect regional differences in capture efficiency, local mutation rates, or genuine biological heterogeneity in mutational processes (Figure 4c).

**Figure 5:**
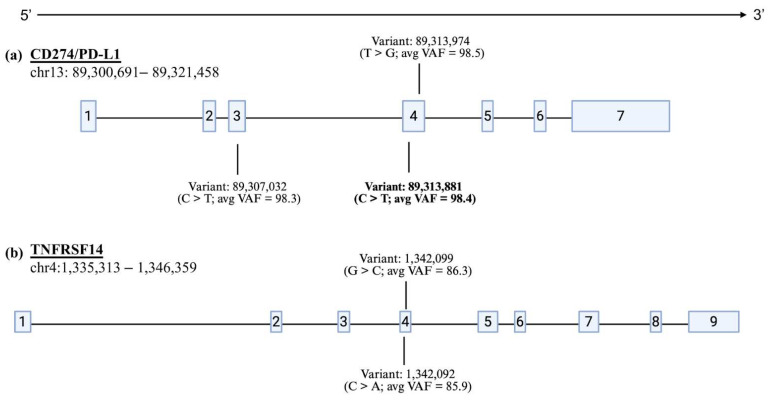
Genes harboring recurrent somatic variants in CSL tumors. Genomic coordinates are shown in base pairs; exons are depicted as boxes. (**a**) *CD274/PD-L1* contained three somatic variants (one in exon 3; two in exon 4. Only the bolded variant was validated by Sanger sequencing. (**b**) Two variants were identified in *TNFRSF14*, both of which were in exon 4 and were unable to be validated with Sanger sequencing.

A filter of TLOD > 6.3 (tumor log odds) was applied, and 4430 unique variants remained. We then looked at variants that were present in more than one individual and fell within an annotated CSL gene. Seventy-four variants (all SNVs) were present in two or more of the eight CSL tumors, and only 26 of these were in an exon. Seventeen of these variants were present in three or more of the eight tumors, with 10 of them located within an exon. Five variants were present in four or more tumor samples and fell within an exon of an annotated gene (Supplementary Table S4); two of which mapped to *TNFRSF14*, and three to *CD274/PD-L1* (Figure 5). All five of these variants had variant allele frequencies (VAFs) above 10% (Supplementary Table S5). The VAF for the remaining SNVs was less than 10% and since this is below the general limit of detection for Sanger sequencing, they were not considered for somatic variant validation.

Surprisingly, only one of the five candidate variants was validated with our molecular assays. This variant (C > T) maps to exon four of the CSL *CD274/PD-L1* gene (CSL chromosome 13: 89,313,881) and was detected via Sanger sequencing of DNA from all eight tumor samples used in WES and was not detected in any of their respective matched-normal samples. In all variant-positive tumors, Sanger sequencing revealed mixed peak signals at the variant position, with both the reference (C) and variant (T) alleles present, and no tumors exhibiting traces consistent with homozygosity for the variant. The variant was also detected in Sanger sequencing traces of the extended CSL cohort.

**Figure 6:**
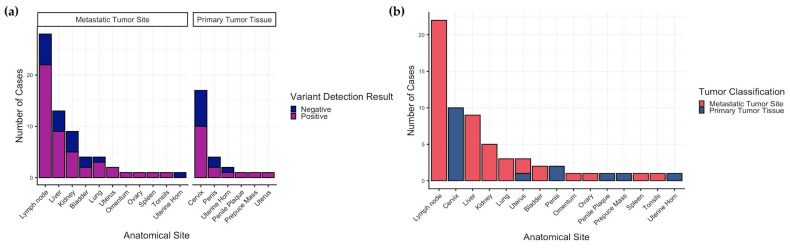
Validation of the recurrent C > T single-nucleotide variant in exon 4 of CD274/PD-L1. Validation was performed by Sanger sequencing of PCR products from an extended cohort of 91 tumors. (**a**) Variant detection by tumor tissue classification (**b**) Total number of positive tumors per anatomical site.

Overall, DNA isolated from 171 specimens from 70 CSLs (56 females, 14 males) were screened for the variant. Sixty-three out of 91 (69.2%) tumor samples screened were positive for the variant. Forty-nine of 72 tumors (68.1%) from female CSLs (*n* = 41 females) were positive, while 14 of 19 tumors (73.7%) from male CSLs (14 males) were positive. Of the 26 samples identified as primary tumors, 16 (61.5%) were positive for the variant. Forty-seven of the 65 (72.3%) samples identified as metastatic tumor tissue sites were positive for the variant (Figure 6a).

To assess within-individual concordance between primary tumors and matched metastatic lesions, we evaluated cases where both sample categories were available for a given individual (Table 2). Variant status was concordant in a subset of individuals and discordant in others, with discordance observed as detection in the primary tumors but not in the matched metastatic lesion.

All anatomical sites represented among tumor samples (primary and metastatic) had at least one positive result (Figure 6b). None of the matched/control normal tissues or whole blood had detectable traces of the variant allele. A full summary of variant screening is shown in Supplementary Table S6.

The mutation results in a synonymous substitution at codon N184 (AAC > AAT). Analysis of genome-wide codon usage in the CSL reference genome revealed that AAC is used more frequently than AAT among asparagine codons (52.72% vs. 47.28%), indicating that AAC represents the genome-wide preferred codon. The recurrent detection of the AAT variant in tumors, despite its lower genome-wide usage, suggests that tumor-specific cellular or translational contexts may permit or favor retention of this otherwise less common codon. However, this analysis does not assess tissue-specific codon usage or imply differential codon preferences across normal tissue types.

**Figure 7:**
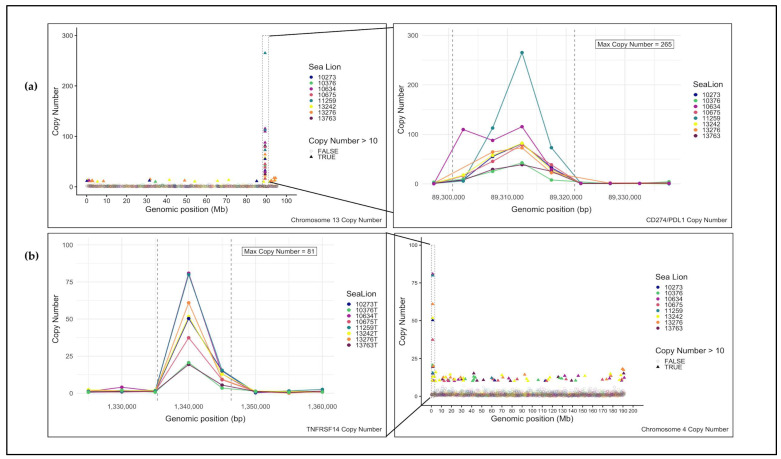
DNA copy number analysis of variant-associated regions from CSL WES data. Both genes with recurrent somatic variants were in regions of significant copy number gain. (**a**) Chromosome-level copy number profiles for the tumors show that the highest gains on chromosome 13 overlap *CD274/PD-L1* (boxed region, with the peak corresponding to exon 4. (**b**) Chromosome 4 profiles reveal recurrent amplifications encompassing *TNFRSF14* (boxed region) (exons 2–7).

All four non-validated variants occurred in genes that exhibited elevated DNA copy number gains in the WES data (Figure 7a,b). The absence of the variant alleles in both tumor and normal Sanger traces indicated these were likely sequencing or amplification artifacts rather than true somatic mutations. Examination of the sequence context surrounding the five candidate variants revealed differences between validated and non-validated sites. The two variants in *TNFRSF14* were located within highly GC-rich regions (~65% GC), while the two non-validated *CD274/PD-L1* variants were in moderately AT-rich regions (~56% AT) and were adjacent to a region of repeat sequences. By contrast, the validated variant was in a region of balanced nucleotide composition (~47% GC) and outside of annotated repetitive elements (Supplementary Table S5).

To assess whether cross-species capture artifacts might contribute to false positive variant calls, we evaluated the distribution of SNVs relative to regional sequence orthology between the dog and CSL. Using the overlap of dog baits with CSL exons and gene annotations from both reference genomes, we classified genomic regions into three categories: high orthology (dog baits mapping to exons annotated as the same gene in both species), reduced orthology (dog baits mapping to exons of differently annotated genes), and no interpretable orthology (baits mapping to unannotated or intergenic regions in either species). This classification reflects genome annotation concordance rather than direct sequence similarity. As expected for a cross-species hybridization system, somatic variant calls were not uniformly distributed across orthology classes. SNV density was significantly higher in regions where dog baits exhibited reduced sequence orthology to the CSL genome (Fisher’s exact test, OR = 1.16, 95% CI:1.04–1.30, *p* = 0.00775). Although the effect was modest, this enrichment suggests that cross-species capture and alignment uncertainty may contribute to a small but detectable increase in regions with discordant gene annotations.

To further investigate the relationship between bait-target orthology and variant authenticity, we examined the orthology classifications of the five candidate variants chosen for orthogonal validation. Of these candidates, two occurred in intervals with no overlap between canine baits and CSL exons (no interpretable orthology), one in a high-orthology exon, and two in reduced-orthology regions. The validated variant was classified as reduced orthology, with the bait mapping to an uncharacterized locus in the dog and *CD274/PD-L1* of the CSL genome. The two variants occurring in non-mapped intervals failed to validate, consistent with these regions representing off target capture or alignment artifacts. The variant in a high-orthology *CD274/PD-L1* exon also failed validation, indicating that somatic variant caller false positives can occur even in well-conserved, confidently mapped regions.

### 3.4. DNA Copy Number Analysis (In Silico)

DNA copy number data for each individual CSL tumor was filtered for DNA copy number ratios above 1 (gain) and below 1 (loss) for comparisons of the tumor to the corresponding matched normal. The dataset was further filtered to only include regions that fell within an exon of an annotated gene on autosomal and sex chromosomes. After this filtering, five genes with apparent gains across all eight tumor exomes were identified: *CD200*, *TNFRSF14*, *CD274/PD-L1*, *CDK4*, *PLCG2* (Table 3). However, the absolute copy number values derived from cross-species exome capture are subject to significant technical artifacts, including uneven hybridization efficiency, potential algorithm misspecification, and the confounding effects of structural variation. These values should be considered preliminary observations that require orthogonal validation before biological interpretation. The consistent direction of changes (gains) across all five genes in all eight tumors, however, suggests a genuine biological pattern of recurrent amplification, though the precise magnitude remains to be confirmed through orthogonal approaches such as fluorescence in situ hybridization (FISH).

## 4. Discussion

In this study, we leveraged resources from the domestic dog to generate comparative DNA sequencing data for somatic variant detection and DNA copy number profiling in the CSL. Our goal was to investigate potential genomic signatures of UGC in the CSL. By employing canine exome capture baits, we sequenced the evolutionarily conserved exome of eight tumor-matched normal tissue pairs from CSLs and demonstrated the efficacy of the cross-species capture system. With these data, we discovered a recurrent SNV, detected in all eight UGC cases, identified small regions of segmental DNA copy number amplification, and found a high prevalence of viral DNA from the oncogenic OtHV-1.

Given the hypothesized association between CSL UGC and OtHV-1, and parallels with human genital cancers linked to oncogenic viruses, we first investigated the prevalence of viral DNA from OtHV-1 in our sample cohort. The detection of OtHV-1 DNA in 95.7% of individuals further supports the hypothesized relationship between this virus and CSL carcinomas [2,25,26]. However, it is important to note that the association between the virus and UGC is limited to the detection of the viral DNA and viral genes in tumor tissues, and no causative links have been established [2,61]. Most samples negative for viral DNA were matched normal tissue and blood samples, though blood samples from individuals with metastatic disease that were positive may reflect the presence of circulating tumor cells or tumor-derived DNA harboring viral sequences. It is important to note that viral DNA was also detected in two of four samples from non-UGC control individuals, but positive detections were limited to blood and urogenital mucosal tissue (cervix), whereas the single control individual without detectable OtHV-1 was represented by only lung and muscle, which are not recognized sites of herpesvirus latency [20,61]. This tissue-specific distribution aligns with the known genital tropism of OtHV-1 and argues against a general or ubiquitous viral presence in non-neoplastic individuals. Detection of viral DNA is histologically normal cervix is consistent with previous work showing that approximately one-third of CSLs with a normal cervix are OtHV-1 positive, but with significantly lower viral loads and absent viral mRNA expression in the epithelium, indicating latent rather than transformative infection [61]. Furthermore, our finding that OtHV-1 DNA was detected significantly more frequently in tumor samples than in non-tumor tissues (*p* = 0.001, OR = 6.70) and remained enriched when tumors were compared to matched normal tissues from affected animals (*p* = 0.003, OR = 6.0), demonstrates that viral presence is markedly concentrated in neoplastic tissue. Mixed-effects logistic regression accounting for multiple tissues per individual confirmed a strong association between tumor status and viral detection (*p* < 0.001). These results are supported by population-level and case–control studies demonstrating that while OtHV-1 infection occurs in a minority of free-ranging CSLs without cancer, the odds of UGC are more than 30–40× higher in OtHV-1 positive animals, indicating a strong association between infection and disease despite some latent infection in clinically normal individuals [17,24]. Thus, the OtHV-1 positive control samples are most consistent with latent genital infection, as described in prior studies, and does not contradict the association between OtHV-1 infection status and UGC in CSLs demonstrated by out statistical analyses and corroborated by prior work.

Several factors may explain the variable detection of OtHV-1 across sample types. Herpesviruses can establish latent infections where the virus persists in a dormant state within tissues without active replication or circulation in the blood producing new viral particles [24,71,72,73]. Additionally, γ herpesviruses (e.g., OtHV-1, EBV, KSHV) exhibit restricted tropism for specific tissues or cell types [74], potentially leading to higher viral loads in preferred tissue types. Methodological limitations may also contribute, as traditional end-point PCR may not detect the virus at loads below the method’s detection threshold. Overall, these findings strengthen the hypothesis that UGC development in the CSL may be induced in part by viral oncogenesis.

Both EBV and KSHV, which exclusively infect humans, are classified as carcinogens and are hypothesized to induce cancer by disrupting normal immune functions [71]. Notably, two of the genes showing amplification or harboring a significant number of somatic mutations in our WES tumor cohort (*TNFRSF14*, *CDK4*) are involved in pathways for herpesvirus infection and viral oncogenesis in humans [75,76,77]. Given the genetic similarity and shared mechanisms of herpesviruses in cellular disruption, OtHV-1 may similarly exploit immune function to promote tumor development.

Four of the five highly recurrent somatic variants identified by Mutect2 did not validate with Sanger sequencing. While initially concerning, this low validation rate provides valuable insight into the technical challenges of cross-species exome sequencing and underscores the critical importance of orthogonal validation in non-model organisms. Somatic variant identification is significantly influenced by bioinformatic tool selection and filtering methods [78], and the intrinsic characteristics of tumor tissues, including elevated genomic instability, higher mutation rates, copy number variations, and structural alterations, introduce substantial challenges for accurate variant calling. Cancer genomes frequently exhibit chromosomal instability and genome reorganization, including translocations, inversions, and complex rearrangements that can disrupt normal gene architecture and complicate read alignment [79,80,81,82]. These structural changes, combined with tumor heterogeneity [78,83,84], pose challenges for variant detection that is further complicated by cross-species capture limitations.

The validation pattern reveals how genome annotation concordance affects variant detection reliability in cross-species applications. The only validated SNV occurred in a reduced-orthology region of *CD274/PD-L1*, where the CSL genome correctly annotates the locus but the corresponding dog bait maps to LOC111096980, an unannotated or provisionally annotated locus in the canine CanFam3.1 reference. However, sequence alignment confirms that CSL codon N184 is orthologous to dog *CD274/PD-L1* N184, indicating that the dog genome contains the orthologous gene at this position, but the RefSeq annotation incorrectly labels it with a provisional LOC identifier. This discrepancy likely reflects differences in gene annotation or exon boundary definitions rather than genuine sequence divergence, as *CD274/PD-L1* exhibits sequence and functional conservation across mammals [85]. Such inconsistencies are common in immune checkpoint genes, which can exhibit rapid evolution in regulatory regions while maintaining conserved protein-coding sequences [86,87]. The successful bait hybridization and variant detection occurred because exome capture depends on DNA sequence conservation, not annotation accuracy, demonstrating that cross-species approaches can reliably identify variants in truly orthologous genes even when reference genome annotations are discordant.

Although our genome-wide analysis showed reduced-orthology intervals were enriched for SNV calls (OR = 1.16, *p* = 0.0078), the successful validation of a functionally important variant in such a region demonstrates that these intervals can harbor genuine somatic mutations and should not be uniformly excluded. Furthermore, because gene naming and annotation quality differ between species and evolve over time, orthology class assignment reflects confidence in the current RefSeq gene models rather than absolute sequence similarity. In cases where reduced orthology reflects annotation errors rather than sequence divergences, as with the validated variant, true biological orthology may be high despite annotation mismatch. As annotations improve, particularly for immune-related and uncharacterized loci, some reduced-orthology intervals may eventually be recognized as true orthologous exons.

In contrast, the four non-validated variants likely arose from distinct technical artifacts. The two *TNFRSF14* variants occurred in intervals with no interpretable orthology between dog and CSL, suggesting off-target bait capture or spurious alignment. One *CD274/PD-L1* variant in a high-orthology exon failed validation despite occurring in a well-conserved region, indicating that false positives can arise independent of cross-species capture issues—likely due to PCR amplification biases, strand-specific sequencing errors, or inflated variant allele fractions in regions with copy number gains [88,89], as represented by the high VAFs seen for the five recurrent variants (Supplementary Table S5).

All five SNVs occurred in genes showing apparent copy number gains, yet only one validated through Sanger sequencing. The co-occurrence of these variants in genes with CNAs suggests that structurally complex genomic regions, whether genuinely amplified or appearing amplified due to technical artifacts, creates systematic challenges for both copy number estimation and variant calling in cross-species capture applications.

The high copy number values observed for these genes likely reflect complex genomic reorganization rather than simple duplications. Aggressive cancers frequently exhibit chromosomal instability arising from processes such as breakage-fusion-bridge cycles, which generate tandem duplications, inverted segments, and chimeric junctions [82,90]. These structural features introduce artifacts into both copy number and variant calling, such that reads from highly amplified regions may align to multiple locations, breakpoint-spanning reads can be misclassified as SNVs, and unequal amplification of alleles may create the appearance of high-frequency variants that are structural artifacts rather than point mutations [91,92,93].

The cross-species capture design compounds these challenges. Divergent sequences between dog and CSL may exhibit reduced or uneven hybridization efficiency, potentially inflating apparent copy number ratios when tumor and normal samples capture with different efficiencies. Additionally, the somatic copy number calling algorithm makes assumptions about read depth distributions and tumor purity that may be violated in cross-species applications where coverage is inherently uneven. The unusually high copy number values and absence of detected shared deletions observed in our data may reflect these technical artifacts introduced from the cross-species approach, uneven hybridization efficiency, or limitations in algorithm assumptions when applied to non-model organisms. We interpret these data as hypothesis-generating evidence for candidate gene prioritization rather than definitive proof of specific copy number values. Future validation with orthogonal methods (e.g., FISH) will be essential to confirm both the presence and magnitude of these copy number alterations and to distinguish biological amplification from technical artifacts.

Local sequence context also contributed to validation failures: non-validated variants were enriched in GC-rich, AT-skewed, or low-complexity regions that reduce capture efficiency and complicate PCR validation [94], whereas the validated variant occurred in a region of balanced nucleotide composition outside annotated repeats. Finally, tumor heterogeneity raises the possibility that alleles present in sequencing libraries were not captured in the DNA subsequently amplified for Sanger sequencing.

The cross-species application of canine exome baits to CSL genomic DNA achieved a 66% on-target rate aligning well with expectations based on the ~40–50 million years of evolutionary divergence between Canidae and Otariidae. Studies using human exome capture reagents on non-human primates have demonstrated that capture efficiency decreases with phylogenetic distance: closely related species (e.g., chimpanzee) show >90% on-target rates, while more divergent species (e.g., macaques) exhibit 70–80% [95,96]. Our observed rate falls within this expected range and demonstrates potential for identifying recurrent alterations in conserved cancer genes, though it introduces incomplete coverage and regional biases that complicate comprehensive variant discovery.

Several principles emerge for interpreting somatic variants from cross-species exome capture. First, reduced-orthology regions can yield true variants, particularly when apparent divergence reflects annotation differences rather than sequence change. These regions warrant caution but should not be fully excluded. In contrast, loci with no interpretable orthology represent the highest risk for alignment artifacts and should be deprioritized. Even high-orthology regions are not immune to false positives, especially with copy number alterations or complex genomic rearrangements, underscoring the importance of orthogonal validation. Finally, uneven capture efficiency across chromosomes highlights how species-specific sequence divergence introduces systematic regional biases.

Despite limitations, cross-species exome capture led to the identification of a recurrent *CD274/PD-L1* variant in 69.2% of CSL tumors, demonstrating that this approach can detect biologically meaningful cancer-associated variants in conserved genes. Future applications would benefit from adjusting variant-calling parameters to reflect reduced coverage certainty, prioritizing candidates from conserved regions, and integrating complementary data types (e.g., RNA-seq, targeted deep sequencing) to strengthen confidence in candidate alterations.

The successfully validated mutation was a synonymous C > T transition at codon N184, located in exon four of *CD274/PD-L1*. While C > T transitions are the most common mutational signature across cancer types [97], typically arising from spontaneous cytosine deamination, the high prevalence of this specific SNV in CSL tumors suggests positive selection rather than neutral drift, especially given its location within a central regulator of tumor immune escape.

Synonymous mutations can be positively selected when they enhance growth and survival by altering translation efficiency under tumor-specific conditions [98,99]. Such effects may arise through changes in mRNA structure, stability, or codon usage optimality [99,100]. Codon usage is not uniform across genomes; instead, certain synonymous codons are used more frequently and are generally translated more efficiently at the genome-wide level [101,102]. Analysis of genome-wide codon usage in the CSL reference genome indicates that the wild-type AAC codon is used more frequently than the variant AAT codon among the asparagine residues (52.72% vs. 47.28%), identifying AAC as the globally preferred codon. The recurrent detection of the AAC > AAT synonymous mutation in CSL tumors despite its lower genome-wide usage, suggests that tumor cells may experience context-dependent selective pressures that permit or favor retention of this otherwise less common codon. This interpretation is consistent with evidence that codon “optimality” can differ between normal and cancer cells due to reprogrammed tRNA pools, altered ribosome composition, and metabolic shifts that reshape translational efficiency [103,104].

This tumor-specific codon optimization hypothesis is supported by the mutation’s location in a key immune checkpoint gene under strong selective pressure during immune evasion. The preferential selection of AAT over AAC in CSL tumors, despite AAC being more common genome-wide, suggests this synonymous change confers a tumor-specific fitness advantage, potentially explaining its enrichment in the *CD274/PD-L1* oncogene [99].

Although *PD-L1* and the *PD-1/PD-L1* pathway exhibit strong overall conservation across mammals [85], the IgV extracellular domain shows evidence of adaptive evolution driven by species-specific immune interactions [86,87]. Cross-species alignment indicates the CSL synonymous variant at codon N184 occurs at the orthologous position in dogs (N184) and humans (K185), with variation in nucleotide sequence across lineages. This pattern suggests that the site tolerates evolutionary flexibility while maintaining overall IgV-domain function. Moreover, functional homology between canine and human *PD-L1* orthologs has been demonstrated across multiple cancer types [105,106,107,108], supporting the biological relevance of the *CD274/PD-L1* mutation identified in CSL tumors, and suggests that immune-evasion mechanisms mediated by this pathway may be broadly conserved across carnivores and other mammalian lineages.

Across the cohort, the *CD274/PD-L1* variant was detected in 61.5% of primary tumor samples and 72.3% of metastatic tumors samples; however, these values represent sample-level detection frequencies rather than independent tumors, as multiple samples were obtained from some individuals. To address this, we evaluated within-individual concordance between paired primary and metastatic tumors. Among the nine individuals with both sample types available, variant status was frequently discordant (55.6%), with all discordant cases showing detection in the primary but not in the matched metastatic lesion. No cases were observed in which the variant was absent in the primary tumor but present in metastatic tissue.

Sanger sequencing of variant-positive tumors consistently revealed mixed signal at the variant position, with both the reference (C) and variant (T) alleles detected, and no samples exhibiting traces consistent with exclusive fixation of the variant (Supplementary Figure S1). However, because Sanger sequencing reflects the average sequence across a heterogeneous mixture of cells, these data do not allow discrimination between true somatic heterozygosity within individual tumor cells, mixtures of homozygous variant and wild-type tumor subclones, or contributions of wild-type sequence from non-tumor cells within the tumor microenvironment. The variable relative peak heights observed across samples nonetheless support substantial intratumoral heterogeneity in variant representation. Tumors lacking detectable variant signal may reflect metastatic lineages derived from variant-negative subclones within the primary tumor. In addition, because Sanger sequencing is unable to reliably detect variants at allele frequencies below ~10–15%, some tumors classified as wild-type may harbor low-frequency variant alleles. Future application of more sensitive approaches, such as digital PCR or single-cell sequencing, will be required to more precisely resolve the prevalence and clonal distribution of the variant.

*CD274/PD-L1* was among five genes (along with *TNFRSF14*, *CDK4*, *PLCG2*, and *CD200*) with copy number gains across all eight tumor exomes. However, these findings must be interpreted cautiously as preliminary observations requiring orthogonal validation. Despite this limitation, the validated *CD274/PD-L1* mutation occurred in a region of apparent amplification, and this convergence of signals provided an additional motivation for focusing validation efforts on this locus. The consistent identification *CD274/PD-L1* across all eight tumors in both variant and copy number analyses suggests that even if absolute copy number values are inflated by technical artifacts, this locus likely harbors genuine alterations.

Overexpression of *CD274/PD-L1* is well documented in human and canine cancers, where it contributes to poor prognosis and is a target for immunotherapy [105,107,109,110,111,112,113]. The *PD-1/PD-L1* axis serves as a mechanism for immune evasion, as *PD-L1* suppresses T-cell activation and allows tumors to develop [105,107,111]. The observed copy number amplification of *CD274/PD-L1* in CSL tumors parallels findings across diverse cancer types, including urothelial carcinoma and cervical carcinoma, where focal gene amplifications activate proto-oncogenes [114,115].

The other four genes identified in the CNA analysis (*TNFRSF14*, *CDK4*, *CD200*, *PLCG2*) remain candidates requiring validation through methods such as qPCR or FISH before their biological significance in CSL UGC can be established. While these genes play established roles in human cancers, including immune regulation, cell cycle control, and viral entry [116,117,118,119,120,121,122,123,124,125,126], we cannot determine from the current data whether they are genuinely amplified in CSL tumors or represent technical artifacts of the cross-species capture approach.

This study provides a methodological framework for investigating somatic genomic changes in genomically underrepresented species by leveraging resources from phylogenetically related species. Key considerations for future applications include: (1) the trade-off between capture efficiency and phylogenetic distance, (2) the importance of stringent variant filtering and orthogonal validation to account for increased technical noise, and (3) the value of focusing analyses on highly conserved cancer-associated genes where probe performance is likely more reliable.

The detection of OtHV-1 DNA in CSL tumor samples supports the hypothesis of viral oncogenesis in this species. The amplification of oncogenes and identification of somatic mutations and CNAs in pathways involved in viral oncogenesis suggest OtHV-1 may play a role in promoting tumor development, similar to oncogenic viruses in human cancer. The discovery of a synonymous mutation and copy number amplification in *CD274/PD-L1* highlights the potential role of this gene in immune evasion strategies conserved across species. The parallels drawn between CSL, human, and canine cancers underscore the importance of comparative approaches and encourage study of spontaneous wildlife cancer for both conservation efforts and advancing human medical research.

## 5. Conclusions

The successful application of canine exome capture reagents to CSL genomic DNA demonstrates the feasibility of leveraging well-characterized model organism resources for comparative oncogenomics in understudied species. Despite ~40–50 million years of evolutionary divergence between Canidae and Otariidae, 66% of dog exon baits successfully targeted annotated CSL exons, with 85.4% of those maintaining gene-level orthology. This cross-species capture efficiency is comparable to rates observed when applying human exome baits to Old World monkeys and suggests that phylogenetic relationships at the mammalian order level may be sufficient for meaningful exome-scale sequencing.

This approach enabled the identification of somatic variants and copy number alterations that would have been prohibitively expensive to discover using species-specific bait design or whole-genome sequencing. However, the 34% off-target rate and potential hybridization biases underscore important limitations when interpreting variant calls and coverage uniformity in cross-species applications.

Our findings contribute to understanding of cancer in the CSL and offer valuable comparative insights for studies of aggressive and highly metastatic cancers in other species. Future studies should continue exploring these connections, utilizing more sensitive and specific techniques to further elucidate the role of viral oncogenesis, somatic mutations, and immune evasion in cancer across species.

## Figures and Tables

**Table 1:** Metadata for all 171 CSL samples analyzed. The CD274/PD-L1 variant (C > T) is located at CSL chr13:89,313,881. “Muscle” denotes unaffected tissue (pectoral). Individuals were categorized as either not having UGC (Control), having carcinoma in situ (CIS), or having metastatic UGC (Met). For some individuals categorized as Met, while metastasis was noted in the pathology report, only the primary tumor tissue was available for this study. * Whole Exome Sequencing Cohort. (a) Metadata for all UGC cases. (b) Metadata for the non-UGC cases.

**(a)**							
**Sea Lion ID**	**Category**	**Sex**	**Sample Type**	**Anatomical Site**	**OtHV-1 DNA Detected**	**Validated CD274 Variant Detected**	**Primary or** **Metastasis**
10273 *	Met	Female	Normal	Muscle	Yes	No	
			Tumor	Liver	Yes	Yes	Metastasis
10376 *	Met	Male	Normal	Muscle	Yes	No	
			Tumor	Bladder	Yes	Yes	Metastasis
10634 *	Met	Female	Normal	Muscle	Yes	No	
			Tumor	Cervix	Yes	Yes	Primary
10675 *	Met	Female	Normal	Muscle	Yes	No	
			Tumor	Kidney	Yes	Yes	Metastasis
11259 *	Met	Female	Normal	Muscle	Yes	No	
			Tumor	Liver	Yes	Yes	Metastasis
13242 *	Met	Male	Normal	Muscle	Yes	No	
			Tumor	Lymph node (Sublumbar)	Yes	Yes	Metastasis
13276 *	Met	Female	Normal	Muscle	Yes	No	
			Tumor	Liver	Yes	Yes	Metastasis
13763 *	Met	Female	Normal	Muscle	Yes	No	
			Tumor	Bladder	Yes	Yes	Metastasis
7755	Met	Female	Normal	Blood	Yes	No	
			Tumor	Lymph node (Sublumbar)	Yes	No	Metastasis
7766	Met	Female	Tumor	Cervix	Yes	Yes	Primary
			Tumor	Kidney	No	No	Metastasis
7778	Met	Male	Normal	Blood	Yes	No	
			Tumor	Lymph node (Iliac)	Yes	Yes	Metastasis
			Tumor	Kidney	Yes	No	Metastasis
7798	Met	Male	Normal	Blood	No	No	
			Tumor	Lymph node (Sublumbar)	Yes	Yes	Metastasis
7909	Met	Male	Normal	Blood	No	No	
			Tumor	Penis	Yes	Yes	Primary
			Tumor	Bladder	Yes	No	Metastasis
			Tumor	Lymph node (Iliac)	Yes	No	Metastasis
7977	Met	Female	Normal	Blood	Yes	No	
			Tumor	Cervix	Yes	No	Primary
			Tumor	Liver	Yes	No	Metastasis
7997	Met	Female	Normal	Blood	Yes	No	
			Tumor	Cervix	Yes	Yes	Primary
			Tumor	Kidney	Yes	No	Metastasis
8059	Met	Female	Normal	Blood	Yes	No	
			Tumor	Cervix	Yes	No	Primary
			Tumor	Uterine Horn	Yes	No	Metastasis
8068	Met	Female	Normal	Blood	No	No	
			Tumor	Cervix	Yes	Yes	Primary
8431	Met	Female	Normal	Blood	No	No	
			Tumor	Cervix	Yes	No	Primary
			Tumor	Liver	Yes	No	Metastasis
9107	Met	Female	Normal	Muscle	Yes	No	
			Tumor	Cervix	Yes	Yes	Primary
9790	Met	Male	Normal	Blood	No	No	
			Tumor	Lymph node (Sublumbar)	Yes	Yes	Metastasis
9853	Met	Female	Normal	Blood	No	No	
			Normal	Uterine Horn	No	No	
			Tumor	Uterine Horn	No	No	Primary
9946	Met	Female	Normal	Blood	Yes	No	
			Tumor	Lymph node (Iliac)	Yes	Yes	Metastasis
			Tumor	Liver	Yes	Yes	Metastasis
9954	Met	Female	Normal	Blood	No	No	
			Normal	Muscle	No	No	
			Tumor	Lymph node (Sublumbar)	Yes	Yes	Metastasis
9975	CIS	Male	Normal	Blood	Yes	No	
			Tumor	Penile Plaque	Yes	Yes	Primary
10161	Met	Female	Tumor	Uterine Horn	No	Yes	Primary
10240	Met	Female	Normal	Muscle	Yes	No	
			Tumor	Cervix	Yes	No	Primary
10272	Met	Female	Normal	Muscle	Yes	No	
			Tumor	Cervix	Yes	Yes	Primary
10281	Met	Male	Normal	Muscle	Yes	No	
			Tumor	Lymph node (Sublumbar)	Yes	Yes	Metastasis
10305	Met	Male	Normal	Muscle	Yes	No	
			Tumor	Lymph node (Sublumbar)	Yes	Yes	Metastasis
10337	Met	Female	Normal	Muscle	Yes	No	
			Tumor	Lymph node (Sublumbar)	Yes	Yes	Metastasis
10395	Met	Female	Normal	Muscle	Yes	No	
			Tumor	Lymph node (Sublumbar)	Yes	Yes	Metastasis
10415	Met	Female	Tumor	Cervix	Yes	No	Primary
10449	Met	Female	Normal	Muscle	Yes	No	
			Tumor	Liver	Yes	No	Metastasis
10482	Met	Female	Normal	Muscle	Yes	No	
			Tumor	Ovary	Yes	Yes	Metastasis
10611	Met	Female	Normal	Muscle	Yes	No	
			Tumor	Lymph node (Hard pigmented)	Yes	No	Metastasis
			Tumor	Lymph node (Unspecified)	Yes	Yes	Metastasis
10725	Met	Female	Normal	Muscle	Yes	No	
			Tumor	Liver	Yes	Yes	Metastasis
10734	Met	Female	Tumor	Cervix	Yes	No	Primary
10749	Met	Female	Normal	Muscle	Yes	No	
			Tumor	Kidney	Yes	Yes	Metastasis
10770	Met	Female	Normal	Muscle	Yes	No	
			Tumor	Lymph node (Sublumbar)	Yes	No	Metastasis
11381	Met	Female	Normal	Muscle	Yes	No	
			Tumor	Liver	Yes	Yes	Metastasis
11613	Met	Female	Normal	Muscle	Yes	No	
			Tumor	Lymph node (Mesenteric)	Yes	Yes	Metastasis
12328	Met	Male	Normal	Muscle	No	No	
			Tumor	Prepuce	Yes	Yes	Primary
12613	Met	Female	Normal	Muscle	No	No	
			Tumor	Cervix	Yes	No	Primary
12871	Met	Female	Normal	Muscle	Yes	No	
			Tumor	Lymph node (Unspecified)	Yes	Yes	Metastasis
13168	Met	Female	Normal	Muscle	Yes	No	
			Tumor	Lymph node (Sublumbar)	Yes	No	Metastasis
13205	Met	Male	Normal	Blood	No	No	
			Normal	Muscle	Yes	No	
			Tumor	Tonsils (hard)	Yes	Yes	Metastasis
13209	Met	Female	Normal	Muscle	Yes	No	
			Tumor	Uterus	Yes	Yes	Primary
13266	Met	Female	Normal	Muscle	Yes	No	
			Tumor	Kidney	Yes	Yes	Metastasis
13281	Met	Female	Normal	Muscle (Site A)	Yes	No	
			Normal	Muscle (Site B)	Yes	No	
			Tumor	Lung	Yes	Yes	Metastasis
13379	Met	Female	Normal	Muscle	Yes	No	
			Tumor	Lymph node (Tracheobronchial)	Yes	Yes	Metastasis
13385	Met	Female	Normal	Muscle	Yes	No	
			Tumor	Lymph node (Mesenteric)	Yes	Yes	Metastasis
13533	Met	Female	Normal	Muscle	Yes	No	
			Tumor	Kidney	Yes	Yes	Metastasis
13621	Met	Female	Normal	Muscle	Yes	No	
			Tumor	Liver	Yes	Yes	Metastasis
13649	Met	Female	Normal	Muscle	Yes	No	
			Tumor	Lymph node (colonic)	Yes	Yes	Metastasis
13664	Met	Female	Normal	Muscle	Yes	No	
			Tumor	Lymph node (Mesenteric)	Yes	Yes	Metastasis
13993	Met	Female	Normal	Muscle	Yes	No	
			Tumor	Lymph node (Tracheobronchial)	Yes	Yes	Metastasis
14314	Met	Female	Normal	Muscle	Yes	No	
			Tumor	Liver	Yes	Yes	Metastasis
14640	Met	Female	Normal	Muscle	No	No	
			Tumor	Lymph node (Pancreatic)	Yes	Yes	Metastasis
14669	Met	Female	Normal	Muscle (Site A)	Yes	No	
			Normal	Muscle (Site B)	Yes	No	
			Tumor	Cervix	Yes	Yes	Primary
Z-21-06-12-046	Met	Female	Normal	Bladder	Yes	No	
			Normal	Cervix	Yes	No	
			Normal	Pancreas	Yes	No	
			Normal	Uterus	Yes	No	
			Normal	Lung	Yes	No	
			Tumor	Bladder	Yes	No	Metastasis
			Tumor	Lymph node (Inguinal)	Yes	Yes	Metastasis
			Tumor	Lymph node (Sublumbar)	Yes	Yes	Metastasis
			Tumor	Lung	Yes	Yes	Metastasis
Z-20-12-10-087	CIS	Male	Tumor	Penis	Yes	No	Primary
Z-20-05-02-044	Met	Female	Normal	Uterus	Yes	No	
			Tumor	Uterus	Yes	Yes	Metastasis
Z-12-01-23-004-R1	Met	Female	Normal	Kidney	Yes	No	
			Normal	Liver	Yes	No	
			Normal	Lung	Yes	No	
			Tumor	Cervix	Yes	Yes	Primary
			Tumor	Kidney	Yes	No	Metastasis
			Tumor	Liver	Yes	No	Metastasis
			Tumor	Lung	Yes	No	Metastasis
Z-23-04-14-027	Met	Female	Normal	Lymph node (Sublumbar)	Yes	No	
			Tumor	Cervix	Yes	Yes	Primary
			Tumor	Lymph node (Sublumbar)	Yes	No	Metastasis
Z-20-05-06-046	Met	Female	Normal	Spleen	Yes	No	
			Normal	Lymph node (Tracheobronchial)	Yes	No	
			Normal	Uterus	Yes	No	
			Tumor	Liver	Yes	Yes	Metastasis
			Tumor	Omentum	Yes	Yes	Metastasis
			Tumor	Spleen	Yes	Yes	Metastasis
			Tumor	Lymph node (Sublumbar)	Yes	Yes	Metastasis
			Tumor	Uterus	Yes	Yes	Metastasis
Z-20-07-07-067	CIS	Female	Tumor	Cervix	Yes	Yes	Primary
Z-22-11-06-088	Met	Male	Normal	Lung	Yes	No	
			Normal	Lymph node (Sternal)	Yes	No	
			Normal	Lymph node (Tracheobronchial)	Yes	No	
			Tumor	Penis	Yes	Yes	Primary
			Tumor	Pelvis	Yes	Yes	Metastasis
			Tumor	Lung	Yes	Yes	Metastasis
Z-22-08-22-078	CIS	Male	Tumor	Penis	Yes	No	Primary
**(b)**							
**Sea Lion ID**	**Category**	**Sex**	**Pathology/Cause of Death**	**Sample Type**	**Anatomical Site**	**OtHV-1 DNA Detected**	**Validated CD274 Variant Detected**
9966	Control	Female	Shark bite trauma; humane euthanasia	Normal	Blood	Yes	No
				Normal	Cervix	Yes	No
13977	Control	Female	Septic arthritis; humane euthanasia	Normal	Muscle	No	No
				Normal	Lung	No	No

**Table 2:** Evaluation of detection results for the validated *CD274/PD-L1* variant (C > T; CSL chr13:89,313,881) in individuals where both primary tumor tissue and metastatic tumor tissue site samples were available. Positive detection is denoted by “+”, and negative detection by “−”. Abbreviations: PN—penis; KD—kidney; LN—lymph node; LU—lung; LV—liver; CX—cervix; UH—uterine horn; BD—bladder.

Sea Lion ID	Primary Tumor	Metastatic Tumor 1	Metastatic Tumor 2	Metastatic Tumor 3
Z-22-11-06-088	PN (+)	KD (+)	LU (+)	NA
7977	CX (−)	LV (−)	NA	NA
8059	CX (−)	UH (−)	NA	NA
8431	CX (−)	LV (−)	NA	NA
7766	CX (+)	KD (−)	NA	NA
7909	PN (+)	BD (−)	LN (−)	NA
7997	CX (+)	KD (−)	NA	NA
Z-12-01-23-004-R1	CX (+)	KD (−)	LV (−)	LU (−)
Z-23-04-14-027	CX (+)	LN (−)	NA	NA

**Table 3:** Shared copy number aberrations detected in the WES data. Regions are based on 5 kb windows; grouped exons fall within these intervals. Values denote copy number estimates per tumor. “-” indicates no deviation from the expected diploid state.

	CD200				TNFRSF14		CD274/PD-L1				PLCG2	CDK4
Sea Lion	Exon 1	Exon 2–3	Exon 4–5	Exon 5	Exon 2–7	Exon 8–9	Exon 1	Exon 2–3	Exon 4	Exon 5–7	Exon 1	Exon 1
10376	3	21	5	-	21	3	8	25	42	8	4	3
13276	4	42	16	9	61	9	-	64	73	23	8	7
13763	6	30	11	5	19	5	8	29	39	25	2	5
10273	6	45	19	9	50	15	9	55	81	33	6	8
10634	4	77	33	10	81	13	110	88	115	30	15	12
13242	6	53	16	4	52	13	18	57	82	29	2	9
10675	3	47	14	5	37	9	16	45	79	38	6	7
11259	10	134	63	7	80	16	6	113	265	73	8	26
Average	6	22	56	5	50	11	25	60	97	32	6	10
Female Average	7	26	62	5	36	11	30	66	109	37	8	11
Male Average	2	11	37	4	55	8	13	41	62	18	3	6

## Data Availability

The original contributions presented in this study are included in the article/Supplementary Materials. Further inquiries can be directed to the corresponding author.

## Supplementary Materials

The following supporting information can be downloaded at: https://doi.org/10.5281/zenodo.18262298, accessed on 1 February 2026. Figure S1: Sanger sequencing traces for the *CD274/PD-L1* variant (CSL chr13: 89,313,881; C > T) for the 8 Tumor/Normal CSL pairs used for WES; Table S1: Histopathology of the eight CSL tumors used for WES; Table S2: WES statistics per sample; Table S3: Average WES statistics per chromosome; Table S4: Recurrent somatic mutations identified with Mutect2; Table S5: Information for the five highly recurrent somatic variants identified by Mutect2; Table S6: Comprehensive detection results for the validated *CD274/PD-L1* variant (CSL chr13:89,313,881); File S1: Merged VCF with variants identified by Mutect2 for all 8 T/N CSL WES pairs.
